# Health related Quality of Life over time in German sarcoma patients. An analysis of associated factors - results of the PROSa study

**DOI:** 10.3389/fendo.2023.1166838

**Published:** 2023-08-29

**Authors:** Martin Eichler, Leopold Hentschel, Susanne Singer, Beate Hornemann, Stephan Richter, Christine Hofbauer, Peter Hohenberger, Bernd Kasper, Dimosthenis Andreou, Daniel Pink, Jens Jakob, Robert Grützmann, Stephen Fung, Eva Wardelmann, Karin Arndt, Kerstin Hermes-Moll, Olaf Schoffer, Marius Fried, Helena K. Jambor, Jürgen Weitz, Klaus-Dieter Schaser, Martin Bornhäuser, Jochen Schmitt, Markus K. Schuler

**Affiliations:** ^1^ Clinic and Polyclinic for Internal Medicine I, University Hospital Carl Gustav Carus, Technical University Dresden, Dresden, Germany; ^2^ National Center for Tumor Diseases Dresden (NCT/UCC), Dresden, Germany; ^3^ German Cancer Research Center (DKFZ), Heidelberg, Germany; ^4^ Faculty of Medicine and University Hospital Carl Gustav Carus, Technical University Dresden, Dresden, Germany; ^5^ Helmholtz-Center Dresden-Rossendorf (HZDR), Dresden, Germany; ^6^ Institute of Medical Biostatistics, Epidemiology and Informatics, University Medical Centre of Johannes Gutenberg University Mainz, Mainz, Germany; ^7^ University Center for Orthopedics and Trauma Surgery, Technical University Dresden, Dresden, Germany; ^8^ Division of Surgical Oncology & Thoracic Surgery, Mannheim University Medical Center, University of Heidelberg, Mannheim, Germany; ^9^ Sarcoma Unit, Mannheim Cancer Center, Mannheim University Medical Center, University of Heidelberg, Mannheim, Germany; ^10^ Department of General Orthopedics and Tumor Orthopedics, Münster University Hospital, Münster, Germany; ^11^ Department of Orthopedics and Trauma, Medical University of Graz, Graz, Austria; ^12^ Sarcoma Center Berlin-Brandenburg, Helios Hospital Bad Saarow, Bad Saarow, Germany; ^13^ Department of Internal Medicine C, University Hospital Greifswald, Greifswald, Germany; ^14^ Clinic for General, Visceral, and Pediatric Surgery, University Hospital Goettingen, Goettingen, Germany; ^15^ Clinic for Surgery, University Hospital Erlangen, Erlangen, Germany; ^16^ Clinic for General, Visceral, and Pediatric Surgery, University Hospital Dusseldorf, Dusseldorfn, Germany; ^17^ Gerhard-Domagk-Institute of Pathology, University Hospital Münster, Münster, Germany; ^18^ German Sarcoma Foundation, Woelfersheim, Germany; ^19^ Scientific Institute of Office-based Hematologists and Oncologists, Cologne, Germany; ^20^ Center for Evidence-based Healthcare, University Hospital Carl Gustav Carus, Technical University Dresden, Dresden, Germany; ^21^ Clinic and Polyclinic for Internal Medicine III/University Cancer Center Mainz, University Hospital Mainz, Mainz, Germany; ^22^ Department of Visceral, Thoracic and Vascular Surgery, University Hospital Carl Gustav Carus, Technical University Dresden, Dresden, Germany

**Keywords:** sarcoma, GIST, health-related quality of life, patient reported outcomes, EORTC QLQ-C30, longitudinal observational cohort

## Abstract

**Introduction:**

Sarcomas are rare cancers and very heterogeneous in their location, histological subtype, and treatment. Health-Related Quality of Life (HRQoL) of sarcoma patients has rarely been investigated in longitudinal studies.

**Methods:**

Here, we assessed adult sarcoma patients and survivors between September 2017 and February 2020, and followed-up for one year in 39 study centers in Germany. Follow-up time points were 6 (t1) and 12 months (t2) after inclusion. We used a standardized, validated questionnaire (the European Organisation for Research and Treatment of Cancer Quality of Life Core Instrument (EORTC QLQ-C30) and explored predictors of HRQoL in two populations (all patients (Analysis 1), patients in ongoing complete remission (Analysis 2)) using generalized linear mixed models.

**Results:**

In total we included up to 1111 patients at baseline (915 at t1, and 847 at t2), thereof 387 participants were in complete remission at baseline (334 at t1, and 200 at t2). When analyzing all patients, HRQoL differed with regard to tumor locations: patients with sarcoma in lower extremities reported lower HRQoL values than patients with sarcomas in the upper extremities. Treatment which included radiotherapy and/or systemic therapy was associated with lower HRQoL. For patients in complete remission, smoking was associated with worse HRQoL-outcomes. In both analyses, bone sarcomas were associated with the worst HRQoL values. Being female, in the age group 55-<65 years, having lower socioeconomic status, and comorbidities were all associated with a lower HRQoL, in both analyses.

**Discussion:**

HRQoL increased partially over time since treatment and with sporting activities. HRQoL improved with time since treatment, although not in all domains, and was associated with lifestyle and socioeconomic factors. Bone sarcomas were the most affected subgroup. Methods to preserve and improve HRQoL should be developed for sarcoma patients.

## 1 Introduction

Sarcomas and gastrointestinal stromal tumors are a group of rare cancers, with about 7000 new cases per year in Germany (1) and an incidence of around 7 per 100,000 in Europe (2). The five-year relative survival in 2010–2016 was 65% for soft tissue sarcomas (STS), 60-79% for bone sarcomas, and 83% for gastrointestinal stromal tumors (GIST) (3). Sarcomas are heterogeneous tumors that include a large variety of over 100 histological subtypes (4), can occur anywhere in the body, and their therapy is based on complex and divergent treatment algorithms (5). Sarcomas are often diagnosed late due to their rarity and the unspecific symptoms they cause (6). Unplanned resections, a result of misdiagnosing the tumors as more common benign or even non-neoplastic lesions, with a negative influence on patient outcome are common (7, 8). Treatment at specialized centers is recommended by international guidelines and is associated with a prolonged survival (9–12).

Cancer patients rate their Health-Related Quality of Life (HRQoL) as an important aspect of their treatment and outcome, and improvement of HRQoL is at times preferred to the mere prolongation of live (13). The multidimensional construct HRQoL itself is a patient-reported outcome, that surveys physical, functional, social, and emotional well-being, as well as disease specific symptoms and restrictions (14).

Literature on HRQoL issues of sarcoma patients has improved over the last few years with two larger studies (15, 16) and a variety of reviews (17–19) published since 2019. Despite these developments, the heterogeneity and rareness of the disease, the variety of treatment pathways experienced by sarcoma patients, as well as the lack of a sarcoma specific measurement tool (20, 21) still leave questions unanswered.

The aim of this analysis was to explore factors associated with longitudinal HRQoL and specifically address lifestyle factors and HRQoL changes over time after the end of treatment. We examined both the predictors of the course of HRQoL in all sarcoma patients regardless of disease stage (Analysis 1) and the predictors of the course of HRQoL in patients in complete remission (Analysis 2).

## 2 Methods

The prospective PROSa cohort study (Burden and Medical Care of Sarcoma in Germany: Nationwide Cohort Study Focusing on Modifiable Determinants of Patient-Reported Outcome Measures in Sarcoma Patients; www.uniklinikum-dresden.de/prosastudie) was conducted nationwide in 39 study centers in Germany between September 2017 and May 2020 (NCT03521531; ClinicalTrials.gov). Of those, 8 were office-based practices, 22 hospitals of maximum care, and 9 other hospitals. Patients were approached at baseline as well as six (t1) and twelve months (t2) after baseline.

Eligible patients and survivors were asked to participate at the study centers during visits (treatment, diagnosis, aftercare) and sometimes by phone or letter. Participation required informed consent. The study was approved by the ethics committees of the Technical University of Dresden (IRB00001473, EK1790422017) and the participating centers (22). Baseline and follow-up data (t1, t2) were collected at the study coordination center at University Hospital Dresden. HRQoL data and socio-demographic data were sent by the participants to the study coordination center by mail or online. In case of non-participation in follow-up, a reminder was sent after 4 weeks. Clinical information was submitted to the study coordination center online by the study centers using case report forms. Data collection was performed using REDCap (23).

We included adult patients and survivors with histologically proven sarcoma of any entity (24). We excluded persons who were mentally or linguistically unable to complete questionnaires. For Analysis 1 (all patients) only participants with HRQoL data were included, for Analysis 2 (patients in ongoing complete remission), we excluded all patients not in complete remission, in current treatment, or with unknown disease status.

### 2.1 Instruments

HRQoL was measured using the European Organisation for Research and Treatment of Cancer Quality of Life Core Questionnaire (EORTC QLQ-C30) (25). This instrument measures global quality of life (global health), 5 functioning, and 9 symptom scales in values from 0 to 100. Higher scores indicate better quality of life for the functioning scales and higher symptom burden for the symptom scales. The relevance of the differences was evaluated using reference values from Cocks et al. (26). With these reference values, each scale difference can be classified as “trivial”, “small”, “medium” and “large”, defined as “Large: one representing unequivocal clinical relevance. Medium: likely to be clinically relevant but to a lesser extent. Small: subtle but nevertheless clinically relevant. Trivial: circumstances unlikely to have any clinical relevance or where there was no difference.” (26).

Socioeconomic status (SES) was assessed using the Winkler Index (27). The Winkler Index is a composite score which covers and quantifies three dimensions of SES: income, education and occupational prestige. On a scale of 3 to 21, a lower score means a lower SES.

The extent of sporting activities was measured using the German Exercise and Sport Questionnaire (“Bewegungs- und Sportfragebogen”) (28). This questionnaire assesses whether patients regularly exercised over the last 4 weeks and asks about the time spent doing so.

Alcohol consumption was grouped in 4 categories (none, weekly or less, regularly moderate (2-3 times a week of up to 4 drinks or 4 times or more a week up to 2 drinks), and regularly larger amounts (more than 4 drinks 2-3 times a week or more than 2 drinks 4 times or more a week.

For Analysis 1 (all patients), we used the patient reported socio-economic variable SES at baseline, as well as the lifestyle variables sporting activities at baseline, t1, and t2 (none, 1-<15 min per week, 15-<30 min per week, ≥30 min per week, unknown), smoking at baseline, t1, and t2 (never, former, actual, unknown), and alcohol consumption at baseline (never, weekly or less, regularly moderate, regularly larger amounts, unknown). From the medical records we collected age at baseline (18-<40, 40-<55, 55-<65, 65-<75, ≥75 years), gender (male, female)the disease characteristics sarcoma type (liposarcoma, fibroblastic/myofibroblastic/fibrohistiocytic sarcoma, GIST, unclassified sarcoma, leiomyosarcoma, bone sarcoma synovialsarcoma, others) and tumor location (abdomen/retroperitoneum, thorax, pelvis, lower limbs, upper limbs, other), comorbidities (cardiovascular, respiratory, diabetes, second cancer, kidney) at baseline (0, 1, 2, ≥3), disease status at baseline, t1, and t2 (complete remission, partial remission/stable disease, progression, unknown), time since treatment at baseline, t1, and t2 (in treatment, <0.5 year, 0.5-<1 year, 1-<2 years, 2-<5 years, ≥5 years, unknown), performed treatments at baseline, t1 and t2 (surgery alone, surgery + systemic therapy (ST), surgery + radiotherapy (RT), surgery + RT + ST, other). For Analysis 2 (patients in ongoing complete remission), we used the above-mentioned variables, except disease status at baseline.

### 2.2 Statistics

Continuous variables were evaluated by mean and standard deviation (SD). Categorical variables were presented with absolute and relative frequencies. We compared participants at all timepoints with those who were lost or did not send back the questionnaire during follow-up to estimate possible selection bias. An analysis of non-participants at baseline was reported elsewhere (16).

For both analyses, five pre-specified domains of the EORTC QLQ-C30 in which the most distinct differences were expected were examined for associated factors: global health, physical functioning, role functioning, pain, and fatigue. We used a generalized linear mixed model with patients as level 1 and timepoint of data collection (baseline, 6 months, 12 months) as level 2.

To avoid multicollinearity, correlations, and tolerance between the model variables were calculated before regression analyses. Correlations ≥ 0.7 and tolerance values ≤ 0.1 indicate strong multicollinearity.

For model variables with more than 5 missing cases, a category “unknown” was created. Imputation method for missing values in SES is described elsewhere (16).

Statistical analyses were performed with SPSS V.28 (IBM Corporation, Armonk, New York, USA).

## 3 Results

### 3.1 Description of the study population/analysis of follow-up non-participants and drop-outs

**Figure 1:**
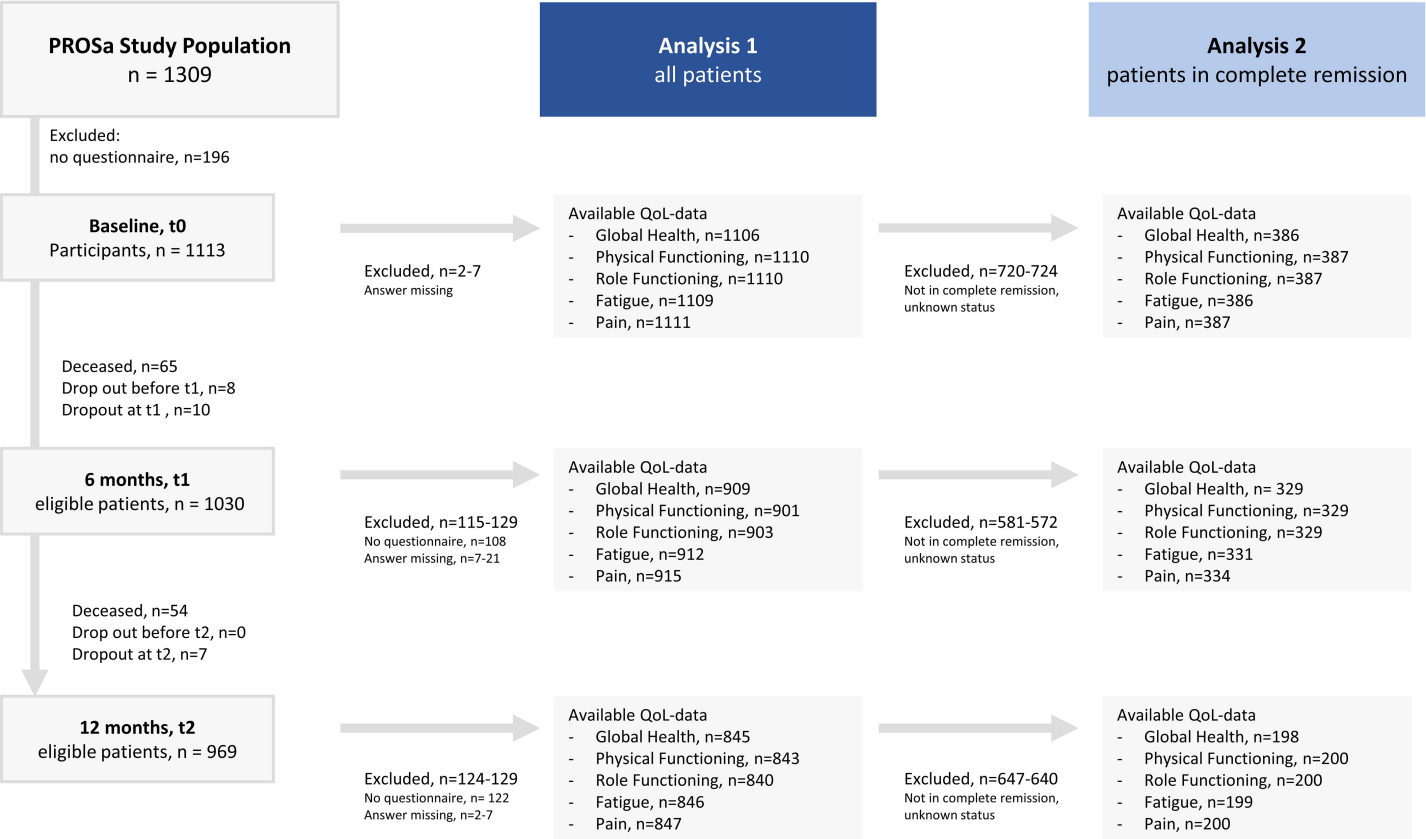
Flow-Chart study population.

In total, 1309 sarcoma patients agreed to participate at baseline, 1030 were eligible at t1, and 969 at t2. Questions on global health were answered by 1106 (baseline), 909 (t1), and 845 (t2) patients. A number of 144 patients dropped out during the study period, 119 of them died. For the Analysis 2 of patients in ongoing complete remission, the general health data was available for 386 (baseline), 329 (t1), and 198 (t2) patients, respectively. See **Figure 1** for a detailed flow-diagram.

A full description of the variables used for Analysis 1 and Analysis 2 are presented in **Table 1**. At baseline, 51% of participants were men, the mean age was 57 years, and around 30% of patients were in treatment. For Analysis 2, 49% of analyzed patients were men, the mean age was 54 years.

**Table 1:** Description of study populations.

Variable	Value	Baseline, all patients, n=1106*		Baseline, patients in complete remission, n= 386*	
		N	%	N	%
Sex **/***	Male	567	51.3	189	49.0
	Female	538	48.7	197	51.0
Age at baseline (18–89)	Mean/Standard Deviation	56.6	15.9	54.4	16.4
Age at baseline **/***	18-<40	185	16.7	82	21.2
	40-<55	262	23.7	90	23.3
	55-<65	297	26.9	99	25.6
	65-<75	230	20.8	85	22.0
	≥75 years	131	11.9	30	7.8
SES at baseline (3–21) **/***	Mean/Standard Deviation	12.8	3.7	12.7	3.6
Sporting activities at baseline **/***	None	711	64.3	205	53.1
	1-<15 min/week	143	12.9	70	18.1
	15-<30 min/week	107	9.7	54	14.0
	≥30 min/week	107	9.7	48	12.4
	Unknown	38	3.4	9	2.3
Smoking at baseline **/***	Never	578	52.3	201	52.1
	Formerly	390	35.3	131	33.9
	Current	128	11.6	48	12.4
	Unknown	10	0.9	6	1.6
Alcohol consumption at baseline **/***	Never	282	25.5	78	20.2
	Weekly or less	541	48.9	202	52.3
	Regularly moderate	242	21.9	87	22.5
	Regularly larger amounts	28	2.5	14	3.6
	Unknown	13	1.2	5	1.3
Sarcoma type **/***	Liposarcoma	210	19.0	72	18.7
	Fibroblastic/myofibroblastic/fibrohistiocytic sarcoma	130	11.8	60	15.5
	GIST	130	11.8	19	4.9
	Unclassified sarcoma	163	14.8	63	16.3
	Leiomyosarcoma	130	11.8	34	8.8
	Bone Sarcoma	205	18.6	90	23.3
	Synovialsarcoma	47	4.3	16	4.1
	Other	88	8.0	32	8.3
Tumor location **/***	Abdomen/retroperitoneum	299	27.0	59	15.3
	Thorax	89	8.0	25	6.5
	Pelvis	160	14.5	44	11.4
	Lower limbs	400	36.2	207	53.6
	Upper limbs	85	7.7	31	8.0
	Other	73	6.6	20	5.2
Time since treatment at baseline **/***	In treatment	365	33.0	–	–
	<0.5 year	79	7.1	27	7.0
	0.5-<1 year	52	4.7	30	7.8
	1-<2 years	84	7.6	59	15.3
	2-<5 years	88	8.0	62	16.1
	≥5 years	80	7.2	59	15.3
	Unknown	358	32.4	149	38.6
Disease status at baseline **	Complete remission	491	44.4	386	100
	Partial remission/stable	328	29.7	-	-
	Progress	160	14.5	-	-
	Unknown	127	11.5	-	-
Received treatments until baseline **/***	Surgery only	291	26.3	146	37.8
	Surgery + ST	285	25.8	82	21.2
	Surgery + RT	162	14.6	89	23.1
	Surgery + RT + ST	232	21.0	69	17.9
	Other	136	12.3	–	–
Number of treatment lines **/***	1 line	592	53.6	293	75.9
	2 or more lines	426	38.5	87	22.5
	unknown	88	8.0	6	1.6
Comorbidities at baseline**/***	0	545	49.3	220	57.0
	1	361	32.6	111	28.8
	2	150	13.6	39	10.1
	≥ 3	50	4.5	16	4.1

*Patients with available HRQoL data on Global Health. **Model variables analysis 1 (baseline). ***Model variables analysis 2 (baseline). ST, systemic therapy; RT, radiotherapy; SES, Socioeconomic Status; GIST, gastrointestinal stromal tumors.

"-" not applicable.

The characteristics of patients surveyed, patients who declined responses, and patients who dropped out during follow-up, differed in parts (**Table 2**). While unadjusted HRQoL values between participants and non-participants were in a range of 2 points, non-participants were on average between 4 and 7 years younger than participants, and more often male than female. Large differences between deceased patients and participants were observed in HRQoL values (ranging from 14 to 20 points) and in gender (overall 50% and 51% of participants were female, among deceased patients this was only 37% and 39%).

**Table 2:** Comparison of Global Health data, sex and age of analyzed (included) patients, unit non responders (no questionnaires) and patients lost to follow up during baseline, 6 months (t1) and 12 months (t2).

	Baseline n	t0 global health mean	Female %	Age at baseline mean	t1 n	t1 global health mean	Female %	Age baseline mean	t2 n	t2 global health mean	Female %	Age at baseline mean
included patients at baseline	1106	59.5	49.7	56.6	-	-	-	-	-	-	-	-
included patients at t1	904	61.1	51.1	57.0	909	61.2	51.2	57.0	-	-	-	-
-deceased until t1	65	39.8	36.9	56.8	–	–	–	–	–	–	–	–
-dropouts before t1	8	46.9	14.3	53.6	–	–	–	–	–	–	–	–
-dropouts at t1	9	47.2	55.6	69.8	–	–	–	–	–	–	–	–
-no questionnaires t1	120	59.7	38.3	52.9	–	–	–	–	–	–	–	–
included patients at t2	840	62.1	50.6	57.1	803	61.8	51.6	57.3	845	62.5	50.7	57.2
-deceased after t1	54	48.0	38.9	59.3	36	49.3	36.1	61.7	–	–	–	–
-dropouts at t2	7	59.5	71.4	61.2	6	62.5	83.3	62.4	–	–	–	–
-no questionnaires t2	123	58.3	46.3	50.5	64	59.9	51.6	50.6	–	–	–	–

Due to changes in item completion, numbers do not always add up to flow-chart numbers. How to read: First column (included patients at baseline): From 1106 patients global health data was available. Second column (included patients t1): From 909 patients with global health data at t1, 904 reported on global health at baseline. Seventh column (included patients at t2): From 845 patients with global health data at t2, 840 reported on global health at baseline and 803 at t1.

"-" not applicable.

### 3.2 Analysis 1: factors associated with the course of HRQoL in all sarcoma patients

Data for Analysis 1 is summarized in **Table 3**, and includes non-standardized regression coefficients (B) indicating a B point increase or decrease in the respective scale. The HRQoL was mostly stable over the observed time, with significant, but trivial differences found in physical functioning, fatigue, and pain. In three domains woman were more strongly affected than men: differences were trivial for physical functioning and pain; small clinically relevant differences were found for fatigue. With younger patients (age: 18-<40 years) as reference value, we observed small but significant differences in all analyzed domains: the most affected groups were patients aged 55-<65 (global health, physical functioning, role functioning and fatigue) and those ≥75 (global health, physical functioning, fatigue, and pain). A 10-point increase in the socioeconomic status was associated with small and significant beneficial changes in global health, physical functioning, fatigue, and pain. Sporting activities were significantly associated with higher HRQoL in all HRQoL domains. Former smokers (comparison: those who never smoked) experienced lower HRQoL in three domains, but those differences were trivial. Compared to patients who consumed no alcohol at all, those with weekly or less consumption or those with a regular, but moderate consumption reported small clinically relevant better outcomes in all domains.

**Table 3:** Factors associated with HRQoL domains over time in German sarcoma patients (Analysis 1).

	Global Health	Physical Functioning	Role Functioning	Fatigue	Pain
Value	B (95% CI)	B (95% CI)	B (95% CI)	B (95% CI)	B (95% CI)
Time point (baseline (ref.))					
t1 (6 month)	-0.7 (-2.1; 0.8)	**-2.8 (-4; -1.6)/^T^**	-0.3 (-2.2; 1.7)	**2.5 (1.0; 4.0)/^T^**	**2.1 (0.2; 3.9)/^T^**
t2 (12 month)	-0.2 (-1.9; 1.5)	**-2.8 (-4.2; -1.4)^/T^**	0.4 (-1.9; 2.6)	**2.8 (1.0; 4.5)/^T^**	1.7 (-0.4; 3.9)
Sex (male (ref.))					
Female	-1.3 (-3.6; 0.9)	**-3.8 (-6.3; -1.3)/^T^**	-2.9 (-6.2; 0.5)	**7.1 (4.2; 10.0)^/S^**	**3.5 (0.1; 6.8)^/T^**
Age at baseline (18-<40 (ref.))					
40-<55	**-6.1 (-9.6; -2.6)/^S^**	**-6.6 (-10.6; -2.6)/^S^**	**-9.5 (-14.8; -4.2)/^S^**	**7.1 (2.5; 11.8)/^S^**	**5.7 (0.3; 11.0)/^T^**
55-<65	**-7.0 (-10.7; -3.3)/^S^**	**-9.0 (-13.2; -4.9)/^S^**	**-10.0 (-15.5; -4.5)/^S^**	**8.0 (3.2; 12.8)/^S^**	3.3 (-2.3; 8.8)
65-<75	-3.2 (-7.2; 0.9)	**-5.6 (-10.2; -1.1)/^S^**	-2.6 (-8.6; 3.4)	-0.1 (-5.4; 5.1)	-1.3 (-7.3; 4.8)
≥75 years	**-6.6 (-11.3; -2.0)/^S^**	**-12.1 (-17.4; -6.9)/^S^**	-5.7 (-12.7; 1.2)	**7.5 (1.4; 13.6)/^S^**	-1.3 (-7.3; 4.8)
SES at baseline					
Per point increase^¶^	**0.7 (0.4; 1.0)/^S^**	**0.7 (0.4; 1.0)/^S^**	0.3 (-0.1; 0.8)	**-0.5 (-0.9; -0.1)^/S^**	**-1.2 (-1.6; -0.7)^/S^**
Sporting activities (none (ref.))					
1-<15 min per week	**6.2 (4.1; 8.3)/^S^**	**4.2 (2.4; 6)/^T^**	**5.4 (2.5; 8.2)/^T^**	**-4.0 (-6.2; -1.7)^/T^**	-2.6 (-5.4; 0.1)
15-<30 min per week	**8.8 (6.4; 11.2)/^S^**	**6.1 (4.0; 8.2)/^S^**	**7.3 (4.0; 10.6)/^S^**	**-6.1 (-8.7; -3.5)/^S^**	**-6.8 (-10.0; -3.7)/^S^**
≥30 min per week	**9.3 (6.8; 11.9)/^S^**	**7.0 (4.8; 9.2)/^S^**	**12.5 (9.1; 15.9)/^S^**	**-8.5 (-11.2; -5.7)/^S^**	**-6.3 (-9.6; -3.0)/^S^**
Unknown	**7.4 (3.9; 11.0)/^S^**	**3.7 (0.7; 6.7)/^T^**	**9.1 (4.2; 13.9)/^S^**	**-6.0 (-9.8; -2.2)/^S^**	**-5.9 (-10.5; -1.3)/^T^**
Smoking (never (ref.))					
Formerly	-2.1 (-4.2; 0.1)	**-2.9 (-5.2; -0.7)/^T^**	**-3.5 (-6.6; -0.3)/^T^**	2.5 (-0.2; 5.1)	**3.9 (0.9; 7.0)/^T^**
Current	-1.1 (-4.3; 2.1)	-2.1 (-5.5; 1.2)	-2.0 (-6.8; 2.7)	1.3 (-2.7; 5.3)	1.9 (-2.8; 6.6)
Unknown	-4.5 (-10.7; 1.7)	-0.1 (-5.3; 5.0)	-0.8 (-9.0; 7.4)	-2.0 (-8.5; 4.5)	-0.6 (-8.5; 7.4)
Alcohol consumption at baseline (Never (ref.))					
Weekly or less	**5.1 (2.5; 7.7)/^S^**	**7.4 (4.5; 10.3)/^S^**	**7.6 (3.7; 11.6)/^S^**	**-6.7 (-10.1; -3.2)/^S^**	**-7.0 (-11.0; -3.1)/^S^**
Regularly moderate	**9.1 (5.8; 12.3)/^S^**	**10.4 (6.7; 14.0)/^S^**	**13.2 (8.3; 18.0)/^S^**	**-9.9 (-14.2; -5.7)^/S^**	**-9.9 (-14.8; -5.0)/^S^**
Regularly larger amounts	-2.0 (-9.0; 5.1)	1.9 (-6.0; 9.8)	3.8 (-6.7; 14.4)	3.9 (-5.3; 13.2)	5.5 (-5.1; 16.1)
Unknown	3.3 (-7.1; 13.7)	7.2 (-4.1; 18.5)	5.1 (-10.1; 20.3)	-7.9 (-21.4; 5.6)	-7.4 (-22.5; 7.8)
Sarcoma Type (liposarcoma (ref.))					
Fibroblastic/myofibro-blastic/fibrohistiocytic s.	-2.5 (-6.5; 1.5)	-1.0 (-5.5; 3.5)	-2.6 (-8.6; 3.4)	2.2 (-3.1; 7.4)	5.4 (-0.7; 11.4)
GIST	1.3 (-3.2; 5.8)	3.2 (-1.9; 8.2)	5.5 (-1.3; 12.2)	0.1 (-5.8; 6.0)	1.5 (-5.3; 8.3)
Unclassified sarcoma	-3.7 (-7.6; 0.2)	**-7.5 (-11.8; -3.2)/^S^**	**-9.2 (-15.0; -3.5)/^S^**	**5.9 (0.8; 10.9)/^S^**	5.6 (-0.2; 11.4)
Leiomyosarcoma.	-0.7 (-4.7; 3.3)	-1.5 (-6.0; 3.0)	-1.6 (-7.5; 4.3)	3.2 (-2.0; 8.4)	-0.7 (-6.7; 5.2)
Bone Sarcoma	**-7.4 (-11.5; -3.2)/^S^**	**-11.5 (-16.2; -6.9)/^S^**	**-15.0 (-21.2; -8.7)/^S^**	**7.7 (2.2; 13.1)/^S^**	**9.7 (3.4; 16.0)/^S^**
Synovialsarcoma	**-7.8 (-13.6; -1.9)/^S^**	-5.6 (-12.1; 1.0)	-8.6 (-17.4; 0.2)	6.5 (-1.1; 14.2)	4.1 (-4.7; 12.9)
Other	-0.3 (-4.6; 4.0)	-3.6 (-8.4; 1.3)	-4.4 (-10.8; 2.1)	4.4 (-1.3; 10.0)	5.2 (-1.3; 11.7)
Tumor location (abdomen/retroperitoneum (ref.))					
Thorax	0.0 (-4.8; 4.8)	3.0 (-2.4; 8.4)	0.1 (-7.1; 7.3)	0.9 (-5.5; 7.2)	-2.6 (-9.9; 4.6)
Pelvis	-3.4 (-7.4; 0.5)	-3.4 (-7.8; 1.0)	-1.2 (-7.1; 4.6)	1.3 (-3.8; 6.5)	5.8 (-0.1; 11.7)
Lower limbs	-2.5 (-6.0; 0.9)	**-5.5 (-9.4; -1.6)/^S^**	**-6.7 (-11.9; -1.5)/^S^**	0.1 (-4.5; 4.6)	**6.6 (1.4; 11.8)/^S^**
Upper limbs	4.5 (-0.5; 9.4)	4.5 (-1.1; 10.0)	-0.8 (-8.1; 6.6)	**-7.0 (-13.5; -0.5)/^S^**	-4.0 (-11.4; 3.5)
Other	-1.5 (-6.7; 3.8)	0.4 (-5.4; 6.3)	-0.2 (-8.0; 7.6)	0.2 (-6.6; 7.1)	0.8 (-7.1; 8.7)
Time since treatment (currently under treatment (ref.))					
<0.5 year	1.5 (-1.0; 4.0)	-1.8 (-4.0; 0.3)	2.0 (-1.5; 5.5)	-2.3 (-5.0; 0.4)	1.5 (-1.8; 4.8)
0.5-<1 year	**4.0 (0.6; 7.4)/^S^**	0.3 (-2.6; 3.2)	**5.4 (0.7; 10.0)/^T^**	-4.5 (-8.1; -0.8)	-0.6 (-5.0; 3.9)
1-<2 years	**5.1 (1.7; 8.4)/^S^**	1.8 (-1.1; 4.8)	**5.5 (0.9; 10.1)/^T^**	**-3.7 (-7.4; -0.05)/^T^**	-1.8 (-6.2; 2.7)
2-<5 years	**7.8 (4.1; 11.5)/^S^**	**3.7 (0.4; 7.1)/^T^**	**9.3 (4.1; 14.5)/^S^**	**-4.5 (-8.6; -0.3)/^T^**	-1.6 (-6.6; 3.4)
≥5 years	**5.9 (1.9; 9.9)/^S^**	**4.6 (0.9; 8.4)/^T^**	**11.3 (5.6; 17.0)/^S^**	**-6.2 (-10.7; -1.6)^/S^**	-2.8 (-8.2; 2.7)
Unknown	**6.0 (3.7; 8.3)/^S^**	**3.1 (1.0; 5.1)/^T^**	**11.3 (8.0; 14.5)/^S^**	**-7.4 (-9.9; -4.8)/^S^**	**-3.3 (-6.4; -0.3)^/T^**
Disease status (complete remission (ref.))					
Partial remission/stable	**-2.6 (-4.9; -0.3)/^T^**	-1.8 (-4.0; 0.3)	**-3.7 (-7.0; -0.5)/^T^**	**3.6 (0.9; 6.2)/^T^**	2.2 (-1.0; 5.4)
Progress	**-6.0 (-8.7; -3.2)/^S^**	**-3.7 (-6.2; -1.2)/^T^**	**-5.7 (-9.5; -1.9)/^T^**	**3.7 (0.6; 6.7)/^T^**	**5.4 (1.7; 9.1)/^T^**
Unknown	-1.6 (-3.8; 0.6)	-1.4 (-3.2; 0.5)	**-4.0 (-7.0; -1.1)/^T^**	1.2 (-1.2; 3.6)	1.2 (-1.7; 4.1)
Received treatments (surgery alone (ref.))					
Surgery + ST	0.7 (-2.3; 3.8)	**-4.8 (-8.1; -1.5)/^T^**	-1.8 (-6.3; 2.7)	3.2 (-0.7; 7.0)	-1.3 (-5.8; 3.2)
Surgery + RT	-0.4 (-3.4; 2.6)	**-4.6 (-7.8; -1.4)/^T^**	-2.3 (-6.8; 2.2)	**7.9 (4.1; 11.6)/^S^**	**6.1 (1.7; 10.5)/^S^**
Surgery + ST + RT	0.3 (-2.8; 3.4)	**-6.0 (-9.3; -2.8)/^S^**	-3.9 (-8.4; 0.6)	**6.9 (3.1; 10.8)/^S^**	2.1 (-2.4; 6.6)
Other	-2.2 (-6.0; 1.7)	**-4.1 (-8.0; -0.3)/^T^**	2.1 (-3.6; 7.7)	**7.0 (2.3; 11.6)/^S^**	4.1 (-1.4; 9.6)
Number of treatment lines (1 (ref.))					
2 or more	**-3.4 (-6.1; -0.7)/^T^**	**-4.0 (-6.9; -1.1)/^T^**	**-7.4 (-11.4; -3.5)/^S^**	**5.6 (2.2; 9.0)/^S^**	**4.3 (0.4; 8.3)/^T^**
unknown	-0.5 (-4.9; 4)	1.6 (-3.3; 6.5)	-4.1 (-10.7; 2.5)	-0.9 (-6.6; 4.8)	4.0 (-2.6; 10.6)
Comorbidities at baseline (none (ref.))					
1	**-2.8 (-5.3; -0.3)/^T^**	**-3.5 (-6.3; -0.7)/^T^**	**-4.6 (-8.4; -0.9)/^T^**	2.9 (-0.4; 6.2)	**4.6 (0.9; 8.4)/^T^**
2	**-4.0 (-7.5; -0.5)/^S^**	**-5.3 (-9.2; -1.3)/^S^**	-4.3 (-9.5; 1.0)	**5.2 (0.6; 9.8)/^S^**	2.5 (-2.8; 7.8)
≥ 3	**-9.2 (-14.7; -3.8)/^S^**	**-13.5 (-19.6; -7.4)/^S^**	**-14.7 (-22.8; -6.5)/^S^**	**14.2 (7.1; 21.3)/^M^**	**9.3 (1.1; 17.5)/^S^**

Results of Generalized Linear Regression Models. B, non-standardized regression coefficient (indicating a B point increase or decrease in the respective scale); GIST, gastrointestinal stromal tumor; ST, systemic therapy, RT, radiotherapy; SES, Socioeconomic Status. 95% CI: 95% confidence interval. T, trivial. S, small. M, medium. L, large differences. ^¶^ Relevance of differences calculated with 10 points.

Bold: significant differences.

Using liposarcoma patients as reference group, bone sarcoma patients were the worst performing group. They showed small clinically relevant and significant differences in all analyzed domains. Patients with unclassified sarcomas experienced worse HRQoL in three (physical functioning, role functioning, fatigue), those with synovialsarcoma in one domain (global health). Patients with tumors located at the lower limbs (reference: abdominal/retroperitoneal sarcomas) reported lower physical and role functioning as well as higher pain. In contrast, patients with tumors located at the upper limbs reported better global health and less fatigue.

With respect to treatment status and time since treatment (reference: patients in treatment), an relevant improvement over time was observed in all domains except pain and physical functioning. Clinically relevant improvements over time were not uniform across domains. For global healtha small clinically relevant improvement was observed 6 months, for role functioning 2 years and for fatigue 5 years after treatment. Differences between patients in complete remission and partial remission/stable disease remained trivial. Patients with progressive disease showed small clinically relevant differences in global health. In comparison to patients treated with surgery alone, those treated additionally with systemic therapy (ST) + radiotherapy (RT) experienced worse physical functioning and more fatigue, and those treated additionally with RT worse fatigue and pain. Patients who received (at least) second line treatment were more affected than those in first line treatment. Small significant differences were observed in role functioning and fatigue. The number of comorbidities was also associated with lower HRQoL for patients with 3 or more comorbidities as the worst performing group over all 5 domains.

### 3.3 Analysis 2: factors associated with the course of HRQoL in patients in ongoing complete remission

Data for Analysis 2 is summarized in **Table 4**. HRQoL stayed largely stable over the observed period, with a significant, but trivial difference found in role functioning. Woman were in three domains more affected than men; significant small clinically relevant differences were found in global health, physical functioning, and fatigue. With younger patients (age: 18-<40 years) as reference, small to medium significant differences were found in all analyzed domains: the most affected groups were patients aged 55-<65 (all domains) and ≥75 (physical and role functioning, fatigue and pain). A 10-point increase in socioeconomic status showed small significant improvements in global health, physical functioning, and pain. Sporting activities were significantly associated with better HRQoL in all HRQoL domains. Current smokers (comparison: never smoked) experienced worse HRQoL in two domains (role functioning and fatigue), those differences were small. Compared to patients who consumed no alcohol at all, those with weekly or less consumption or those with a regular, but moderate consumption reported small clinically relevant better outcomes in all domains.

**Table 4:** Factors associated with HRQoL domains over time in German sarcoma patients in complete remission (analysis 2).

	Global Health	Physical Functioning	Role Functioning	Fatigue	Pain
Variable/ Value	B (95% CI)	B (95% CI)	B (95% CI)	B (95% CI)	B (95% CI)
Time point (baseline (ref.))					
t1 (6 month)	0.1 (-1.9; 2.1)	-0.8 (-2.5; 0.8)	1.8 (-1.0; 4.5)	0.5 (-1.6; 2.5)	1.5 (-1.1; 4.2)
t2 (12 month)	1.3 (-1.2; 3.7)	-0.9 (-2.8; 0.9)	**3.5 (0.3; 6.8)/^T^**	0.3 (-2.3; 2.9)	-0.8 (-4.2; 2.6)
Sex (male (ref.))					
Female	**-4.4 (-8.2; -0.6)/^S^**	**-7.3 (-11.4; -3.1)/^S^**	-4.7 (-10.6; 1.1)	**10.1 (5.2; 15.1)^/S^**	5.5 (-0.1; 11.1)
Age at baseline (18-<40 (ref.))					
40-<55	**-5.7 (-11.3; -0.01)^/S^**	**-8.7 (-14.8; -2.6)/^S^**	**-12.4 (-21.1; -3.7)/^S^**	**10.7 (3.3; 18.0)/^S^**	**10.4 (2.1; 18.7)/^S^**
55-<65	**-8.7 (-14.6; -2.7)/^S^**	**-12.4 (-18.8; -5.9)/^S^**	**-17.8 (-27.0; -8.6)/^S^**	**14.7 (6.9; 22.5)/^M^**	**11.2 (2.5; 20.0)/^S^**
65-<75	-1.9 (-8.7; 4.9)	-6.8 (-14.1; 0.5)	-9.9 (-20.3; 0.5)	5.0 (-3.8; 13.9)	8.3 (-1.7; 18.2)
≥75 years	-8.8 (-18.1; 0.6)	**-20.0 (-30.0; -9.9)/^M^**	**-17.2 (-31.4; -2.9)/^S^**	**15.7 (3.6; 27.9)/^M^**	**14.7 (1.1; 28.3)/^M^**
SES at baseline					
Per point increase^¶^	**0.5 (0.02; 1.1)/^S^**	**0.8 (0.2; 1.3)/^S^**	0.6 (-0.2; 1.4)	-0.6 (-1.3; 0.1)	**-1.1 (-1.9; -0.3)^/S^**
Sporting activities (none (ref.))					
1-<15 min per week	**6.1 (2.9; 9.4)/^S^**	2.3 (-0.3; 4.9)	1.0 (-3.4; 5.3)	-2.0 (-5.4; 1.4)	1.7 (-2.6; 6.0)
15-<30 min per week	**7.3 (3.8; 10.8)/^S^**	**4.5 (1.7; 7.3)/^T^**	4.1 (-0.6; 8.8)	**-4.5 (-8.2; -0.8)/^S^**	-3.6 (-8.2; 1.1)
≥30 min per week	**7.1 (3.4; 10.8)/^S^**	**6.4 (3.4; 9.3)/^S^**	**8.9 (4.0; 13.9)/^S^**	**-6.6 (-10.5; -2.7)^/S^**	**-6.1 (-11; -1.2)/^S^**
Unknown	2.7 (-3.2; 8.6)	1.5 (-2.9; 6.0)	5.1 (-2.5; 12.8)	-2.9 (-8.9; 3.0)	-3.9 (-11.5; 3.7)
Smoking (never (ref.))					
Formerly	0.4 (-3.3; 4.0)	-1.9 (-5.5; 1.7)	-2.0 (-7.4; 3.4)	3.7 (-0.8; 8.1)	-0.8 (-6.0; 4.4)
Current	-2.5 (-7.9; 3.0)	-5.3 (-10.7; 0.1)	**-10.0 (-18.2; -1.9)/^S^**	**9.3 (2.5; 16.0)/^S^**	-2.9 (-10.8; 4.9)
Unknown	-3.8 (-13.2; 5.5)	3.5 (-3.2; 10.2)	4.5 (-7.1; 16.2)	-3.7 (-12.9; 5.6)	-3.6 (-14.9; 7.8)
Alcohol consumption at baseline (Never (ref.))					
Weekly or less	**6.1 (1.3; 10.8)/^S^**	4.3 (-0.9; 9.5)	**7.5 (0.1; 14.8)/^S^**	**-7.8 (-14.0; -1.6)/^S^**	**-7.3 (-14.3; -0.3)^/S^**
Regularly moderate	**8.7 (2.8; 14.7)/^S^**	**7.3 (0.9; 13.7)/^S^**	**14.5 (5.5; 23.6)/^S^**	**-11.9 (-19.6; -4.2)/^S^**	**-10.5 (-19.2; -1.8)/^S^**
Regularly larger amounts	-5.6 (-16.1; 4.9)	-1.7 (-13.2; 9.7)	3.5 (-12.7; 19.7)	2.9 (-10.9; 16.6)	1.1 (-14.4; 16.6)
Unknown	-0.1 (-18.8; 18.5)	2.4 (-16.0; 20.7)	14.5 (-11.8; 40.8)	-15.7 (-39.9; 8.5)	-17.8 (-42.7; 7.1)
Sarcoma Type (liposarcoma (ref.))					
Fibroblastic/myofibro-blastic/fibrohistiocytic s.	-1.6 (-7.9; 4.7)	0.1 (-6.7; 7.0)	1.5 (-8.2; 11.3)	1.7 (-6.5; 9.9)	-0.2 (-9.5; 9.1)
GIST	-1.1 (-11.6; 9.4)	5.7 (-5.8; 17.1)	7.1 (-9.0; 23.2)	-2.0 (-15.7; 11.8)	-6.7 (-22.1; 8.7)
Unclassified sarcoma	-3.1 (-9.5; 3.3)	-6.2 (-13.1; 0.7)	-4.8 (-14.5; 4.9)	5.3 (-3.0; 13.6)	7.3 (-2.0; 16.6)
Leiomyosarcoma.	1.3 (-6.2; 8.7)	6.8 (-1.4; 14.9)	4.4 (-7.1; 15.8)	-3.0 (-12.7; 6.8)	-7.6 (-18.6; 3.3)
Bone Sarcoma	**-7.6 (-14.4; -0.9)/^S^**	**-9.4 (-16.8; -2.0)/^S^**	**-12.1 (-22.5; -1.7)^/S^**	7.4 (-1.4; 16.3)	7.1 (-2.9; 17.0)
Synovialsarcoma	-2.2 (-12.3; 8.0)	-3.6 (-14.6; 7.4)	1.1 (-14.5; 16.7)	1.1 (-12.1; 14.3)	2.2 (-12.7; 17.0)
Other	1.0 (-6.9; 8.9)	-1.3 (-9.8; 7.2)	1.5 (-10.6; 13.6)	1.9 (-8.3; 12.1)	-1.4 (-13.0; 10.1)
Tumor location (abdomen/retroperitoneum (ref.))					
Thorax	2.8 (-6.5; 12)	2.6 (-7.4; 12.7)	1.8 (-12.5; 16.0)	-0.3 (-12.3; 11.8)	-12.0 (-25.6; 1.6)
Pelvis	-0.1 (-8.2; 7.9)	-4.6 (-13.3; 4.1)	-0.8 (-13.1; 11.5)	1.3 (-9.2; 11.7)	3.9 (-7.9; 15.6)
Lower limbs	-0.1 (-6.7; 6.6)	-6.5 (-13.7; 0.7)	-4.5 (-14.7; 5.8)	1.3 (-7.4; 9.9)	0.6 (-9.2; 10.3)
Upper limbs	4.3 (-4.7; 13.3)	-1.2 (-10.9; 8.6)	-2.9 (-16.8; 11.0)	-0.9 (-12.7; 10.8)	-10.3 (-23.6; 3.0)
Other	0.2 (-9.9; 10.4)	-5.8 (-16.8; 5.2)	-0.6 (-16.2; 15.0)	7.0 (-6.3; 20.3)	3.7 (-11.2; 18.6)
Time since treatment (<0.5 year (ref.))					
0.5-<1 year	5.7 (-0.9; 12.3)	4.8 (-0.4; 10.1)	7.5 (-1.7; 16.6)	-3.7 (-10.6; 3.3)	-5.8 (-14.5; 2.9)
1-<2 years	**7.9 (1.3; 14.5)/^S^**	5.4 (-0.04; 10.8)	8.4 (-1.0; 17.8)	-0.5 (-7.8; 6.8)	-2.6 (-11.7; 6.5)
2-<5 years	**8.6 (1.4; 15.9)/^S^**	**6.7 (0.6; 12.8)/^S^**	6.6 (-3.8; 17.0)	-0.6 (-8.7; 7.4)	1.7 (-8.2; 11.6)
≥5 years	**10.7 (2.9; 18.6)/^M^**	**8.9 (2.0; 15.8)/^S^**	**12.4 (1.1; 23.8)/^S^**	-4.5 (-13.4; 4.4)	-2.7 (-13.4; 8.0)
Unknown	**8.5 (0.9; 16.1)/^S^**	**4.9 (-1.8; 11.6)/^T^**	**11.7 (0.7; 22.7)/^S^**	-2.6 (-11.2; 6.0)	-2.6 (-13.0; 7.8)
Received treatments (surgery alone (ref.))					
Surgery + ST	-1.4 (-6.7; 3.9)	-4.6 (-10.3; 1.1)	-4.7 (-12.9; 3.4)	4.2 (-2.7; 11.1)	1.0 (-6.8; 8.7)
Surgery + RT	-1.6 (-6.9; 3.8)	2.3 (-3.5; 8.0)	2.9 (-5.2; 11.1)	2.5 (-4.4; 9.4)	0.7 (-7.0; 8.5)
Surgery + ST + RT	2.4 (-3.3; 8.1)	2.2 (-4.0; 8.4)	-0.7 (-9.5; 8.0)	3.6 (-3.9; 11.0)	-4.4 (-12.8; 3.9)
Number of treatment lines (1 (ref.))					
2 or more	0.5 (-5.0; 6.0)	-1.1 (-6.8; 4.6)	-5.2 (-13.5; 3.2)	1.0 (-6.0; 7.9)	5.4 (-2.6; 13.4)
unknown	6.2 (-9.4; 21.7)	-1.3 (-17.9; 15.3)	-8.6 (-32.4; 15.2)	-0.1 (-20.1; 19.9)	-8.5 (-31.0; 14.1)
Comorbidities at baseline (none (ref.))					
1	-2.9 (-7.3; 1.5)	-4.3 (-9.1; 0.5)	-5.8 (-12.5; 1.0)	4.4 (-1.3; 10.1)	4.2 (-2.3; 10.6)
2	**-6.6 (-13.3; 0.2)/^S^**	**-8.0 (-15.3; -0.6)/^S^**	-0.8 (-11.2; 9.6)	6.3 (-2.6; 15.1)	-5.8 (-15.8; 4.1)
≥ 3	-7.5 (-17; 2.0)	**-11.7 (-22.0; -1.3)/^S^**	-10.3 (-24.9; 4.2)	12.1 (-0.3; 24.5)	5.3 (-8.6; 19.2)

Results of Generalized Linear Regression Models. B, non-standardized regression coefficient (indicating a B point increase or decrease in the respective scale); GIST, gastrointestinal stromal tumor. ST, systemic therapy, RT, radiotherapy; SES, Socioeconomic Status. 95% CI: 95% confidence interval. T, trivial. S, small. M, medium. L, large differences. ^¶^ Relevance of differences calculated with 10 points.

Bold: significant differences.

Compared to liposarcoma patients as reference, patients with bone sarcoma were the worst performing group. They showed small clinically relevant and significant differences in general health, physical and role functioning. Other differences did not reach significance. No significant differences were observed with regard to tumor location.

With respect to time since treatment (reference: up to 6 months after treatment) an improvement over time was observed in all domains except pain and fatigue. Improvement over time was not uniform across domains and took longest in role functioning, reaching clinical relevance in this domain 5 years after treatment. In global health, a small clinically relevant improvement was observed 12 months after treatment and a medium improvement after 5 years. In physical functioning a small clinically relevant improvement was observed 2 years after treatment.

No significant differences were observed for the received treatment or the number of treatment lines. The number of comorbidities was associated with lower HRQoL experiences in general health and physical functioning.

## 4 Discussion

### 4.1 Results in context

Our study extends previous quality of life research in oncology through the analysis of a large national sample of sarcoma patients with standardized follow-up over a 1-year period. As expected, in our heterogeneous population of sarcoma patients, differences were found between factors studied in both longitudinal analyses.

The observed differences between gender and age-groups in Analyses 1 and 2 are reported in almost all HRQoL studies in cancer populations (29). It is noteworthy that patients between 55-<65 years (in addition to those ≥75) represented the most restricted age group. This implies that reaching retirement age is accompanied by a temporary improvement in various quality-of-life domains, possibly associated with retirement itself, an aspect we previously reported in more detail for a cross sectional analysis of the data (16). Differences between women and men seemed to be larger in patients in complete remission compared to all patients. The association between HRQoL and SES was already observed in the aforementioned study, indicating that a holistic approach to health always should include the social-economic situation of patients.

To our knowledge, there is little sarcoma specific literature on the relation of HRQoL and the analyzed lifestyle factors sporting activities (30, 31), smoking, and alcohol consumption, even if the importance of maintaining physical activity is discussed (32–34). Sporting activities were in both analyses and across all domains associated with improved HRQoL. In the literature about cancers in general, this observation is well established (35, 36). However, our study design – even if longitudinal – does not allow causal conclusions, as it remains possible that a better HRQoL enables sporting activities and is not the result of it. This may be particularly obvious in the case of role functioning (fulfilling everyday tasks), which can conceptually include sporting activities. In the analysis of all patients, former smoking, but not current smoking, was associated with deteriorated HRQoL. It might be the case, that decreased HRQoL lead to smoking cessation. In the analysis of patients in complete remission, however, current smoking was associated with poorer HRQoL. This association is well established in the literature (37–39).

Moderate alcohol consumption was associated with an improvement in HRQoL compared to no consumption in both analyzed groups. This at first glance counterintuitive result is observed in other studies (40–42) and likely reflects the fact, when alcohol consumption is widespread and socially acceptable as is the case for Germany (43), many of those who do not consume alcohol (anymore) may be doing so because they are too ill or weak to drink alcohol. So here we might observe reverse causality, too.

Exploring the relation between time since treatment and analyzed HRQoL domains, a heterogenous picture was revealed. While in Analysis 1 (all patients), a rapid improvement over time was observed for general health, for role functioning, and fatigue clinically relevant improvements were observed only 2 resp. 5 years post treatment, while pain did not improve at all significantly and physical functioning not in a clinically relevant manner. In Analysis 2 (complete remission), HRQoL did not improve regarding pain and fatigue and improved at different times in general health, physical, and role functioning. This ambiguous picture is somewhat reflected in the literature, where for different populations some studies reported improvements 1 year after treatment (44, 45), while others reported more stable trajectories (46, 47). As the study population in the complete remission analysis is more homogenous than in the all-patients analysis, conclusions are probably easier to draw from the former one. Here, the stable trajectory in pain and fatigue raises the question, whether there is room for improvement especially in follow-up care (48, 49).

In Analysis 1, the heterogeneity of sarcoma patients was reflected in the analysis of subgroups and tumor location (15, 16). In Analysis 2, those differences did not remain statistically significant, which might be, at least partly a result of the smaller sample size. The exception are bone sarcoma patients, which were in both analyses stronger affected by functional impairments and lower general health than liposarcoma patients. Van Eck et al. reported that patients with sarcomas of the axial skeleton were the most affected group, a location that we did not document separately (15).

Patients treated with surgery alone had the lowest restrictions in terms of functional impairment and symptom burden in the all-patients analysis. Patients treated with surgery + ST + RT were most affected in all other domains, with the exception of pain, where patients with surgery + RT reported strongest symptoms. Interestingly, no differences were observed in the complete remission cohort. Winnette et al. reported increased burden after CT and RT, as well (50). Further, van Eck et al. compiled a comprehensive list of problems associated with specific treatments (15). Van Tine et al. observed a relatively rapid decline in HRQoL during doxorubicin-based treatment (51). An ongoing observational cohort study is investigating effects of CT on HRQoL (52).

As expected, progressive disease and the number of comorbidities were significantly and relevantly associated with most domains (53).

Overall, our research extends the existing knowledge on the HRQoL in sarcoma patients by providing a comprehensive and longitudinal analysis of a diverse national sample. Moreover, the inclusion of lifestyle factors, socio-economic status, the examination of time since treatment and disease-related factors add valuable insights to the current understanding of HRQoL in this patient population. We were thus able to capture a more holistic picture of the disease.

### 4.2 Strength and limitations

The PROSa study is one of the largest studies on HRQoL in sarcoma patients and survivors worldwide. Patients from 39 German hospitals and practices were included, representing a broad spectrum of sarcoma treating facilities and disciplines. We were able to follow patients for one year and thus to collect and analyze longitudinal data. The analysis has a limitation in the heterogenous patients collective. Despite being able to collect information about a relatively large number of patients, heterogeneity of the disease makes a subgroup analysis difficult. We were not able to compute interaction-analyses, especially on potential different HRQoL-trajectories in sarcoma subgroups (type, location, or treatment).

Although the present study has a longitudinal design, causal conclusions should be drawn cautiously. As discussed with respect to sporting activities, changes in HRQoL outcomes may have occurred before changes in independent variables. Unobserved or spurious confounding is also possible and the study is subject to selection bias. The majority of our patients were recruited in university hospitals and/or specialized centers and thus might not be representative for all sarcoma patients. In addition, we suspect a sick survivor bias, as healthy survivors have less frequent contact with our recruiting study centers. For Analysis 1, it should additionally be considered, that, as patients were in most cases recruited during hospital visits, the probability that the course of HRQoL over time is influenced by a subsequent treatment or worsening of disease is higher than in a sarcoma population recruited at a random timepoint. Our study population changed over the course of one-year, with younger patients and men being overrepresented in non-participants during follow-up, and there is the possibility that this influenced our results.

## 5 Conclusion

The heterogeneity of sarcomas regarding type, location, and treatment is reflected in the HRQoL outcomes in the analysis of all sarcoma patients, but only to a certain extent in the complete remission cohort. Bone sarcoma patients were in both analyses the most affected sarcoma type, while significant differences regarding location (patients with tumors located at the lower extremities performed worst and those with sarcomas at the upper extremities best) and treatment (having received radiotherapy and/or systemic therapy was associated with lower HRQoL in some domains) were only found in the analysis of all patients.

In both cohorts, sociodemographic factors like age, sex, and socioeconomic status were associated with HRQoL as well. Patients between 55-<65 years of age were the most affected group, indicating that certain life circumstances, like work, may increase the disease burden.

Lifestyle factors, especially sporting activities were strongly associated with HRQoL outcomes. Additionally, smokers had a higher probability of experiencing deteriorated HRQoL scores. It is thus important that clinicians are aware of these factors and take them into account and address them during treatment and follow-up. They should be considered in the development of supportive care programs.

Outcomes improved in both analyses over time since treatment, albeit in different patterns. Patients in ongoing complete remission improved over time in global health, physical, and role functioning. Such improvements were not observed for fatigue and pain. These findings can inform healthcare professionals and policymakers in developing targeted interventions and support services to enhance the well-being and overall quality of life for sarcoma patients.

## Data availability statement

The raw data supporting the conclusions of this article will be made available by the authors, without undue reservation.

## Ethics statement

The studies involving humans were approved by Ethics committees of the Technical University of Dresden. The studies were conducted in accordance with the local legislation and institutional requirements. The participants provided their written informed consent to participate in this study.

## Author contributions

ME wrote the article and analyzed the data. ME, MS, and LH developed questionnaires and study design. JS and MS developed the conception of the study and supervised with MB the work throughout the whole study. EW supervised development of inclusion criteria. KA supervised the study from a patient’s perspective. ME and SS developed the statistical analysis plan for this paper. OS supervised statistical analysis. HJ did scientific editing and developed visualizations. SR, CH, PH, BK, DA, DP, JJ, RG, SF, KH-M, MF, JW, K-DS, and MS were responsible for the recruitment of patients or recruited patients directly. All authors have revised the manuscript critically and approved the published version.
